# Mapping the use of patient reported outcomes at a health organisation to inform integration into hospital data systems.

**DOI:** 10.23889/ijpds.v7i3.1883

**Published:** 2022-08-25

**Authors:** David Snowdon, Velandai Srikanth, Lucy Marsh, Richard Beare, Emily Parker, Nadine Andrew

**Affiliations:** 1 National Centre for Healthy Ageing / Monash University

## Objectives

Incorporating patient reported outcomes into health data linkage research ensures that the patient’s perspective is considered. We aimed to understand the use of patient reported outcomes across an entire healthcare organisation, to inform routine collection and integration of patient reported outcomes into hospital data systems for clinical practice and research.

## Approach

We applied a mapping process consisting of 1) an audit of patient reported outcomes used in research projects and data registries, and 2) a survey of clinicians’ use of patient reported outcomes in their clinical practice from January 2015 to March 2021. Patient reported outcomes were then classified as ‘specific’ to a particular disease and/or conditions, or as a ‘generic’ measure that was applicable to a broader population. Patient reported outcomes were also mapped to the health domains they measured, using the World Health Organisation International Classification Framework. Data were described using frequency and proportion.

## Results

Patient reported outcomes were used by 22% of research projects (n=144/666), 68% of clinical registries (n=13/19), and 76% of clinical specialties in their clinical care (n=16/21). Of the projects, registries and specialties that used patient reported outcomes, disease specific outcomes were most commonly used: 83% for research projects n=130/144), 69% for clinical registries (n=9/13), and 75% for clinical specialties (n=12/16). Greater than 80% of research projects, clinical registries and clinical specialties measured health domains relating to both body impairments and participation in daily life activities. The most commonly used generic patient reported outcome for research, data registries and clinical practice was the European Quality of Life Five Dimension Five Level (EQ-5D-5L) (research projects n=31/144, 22%; data registries n=2/13, 15%; clinical specialties n=3/16, 19%).

## Conclusion and Relevance

In our setting, the EQ-5D-5L had broad applicability across a range of clinical specialties, collection systems and research studies, making it suitable for routine integration into health data systems with potential for linkage with other population data. Sufficient flexibility is required for the collection of population and disease specific measures.

